# The Role of Limited English Proficiency and Access to Health Insurance and Health Care in the Affordable Care Act Era

**DOI:** 10.1089/heq.2020.0057

**Published:** 2020-12-11

**Authors:** Andriana M. Foiles Sifuentes, Monica Robledo Cornejo, Nien Chen Li, Maira A. Castaneda-Avila, Jennifer Tjia, Kate L. Lapane

**Affiliations:** ^1^ Department of Population and Quantitative Health Sciences, University of Massachusetts Medical School, Worcester, Massachusetts, USA.; ^2^ Department of Anthropology, Sonoma State University, Rohnert Park, California, USA.

**Keywords:** English proficiency, insurance, Latino, access

## Abstract

**Purpose:** Limited English proficiency adversely impacts people's ability to access health services. This study examines the association between English language proficiency and insurance access and use of a usual care provider after the implementation of the Affordable Care Act (ACA).

**Methods:** Using cross-sectional data from the 2016 Medical Panel Expenditures Survey, we identified 24,099 adults (weighted *n*=240,035,048) and categorized them by self-reported English-language proficiency. We classified participants according to responses to: “How well do you speak English? Would you say… Very well; well; Not well; Not at all?” (having limited English proficiency: not well; not at all, English proficient: well; very well; and English only: not applicable) and “What language do you speak at home? Would you say… English, Spanish, Other.” Using these two recoded variables, we created a variable with five categories: (1) Spanish speaking, with limited English proficiency, (2) other language speaking, with limited English proficiency, (3) Spanish speaking, English proficient, (4) other language speaking, English proficient, and (5) English only. Health insurance and usual care provider were determined by self-report.

**Results:** Among those <65 years, the percent covered by public insurance (Spanish: 21%, Other languages: 28%, English only 14%), who were uninsured (Spanish: 46%, Other languages: 17%, English only: 8%), and who lacked a usual care provider (Spanish: 45%, Other languages: 35%, English only: 26%) differed by English language proficiency. Among those ≥65 years, fewer people with limited English proficiency relative to English only were dually covered by Medicare and private insurance (Spanish: 12%, Other languages: 15%, English only: 59%), and a higher percent lacked a usual care provider (Spanish: 15%, Other languages: 11%, English only: 7%). Differences persisted with adjustment for covariates.

**Conclusion:** Post the ACA, persons with limited English proficiency remain at a risk of being uninsured relative to those who only speak English.

## Introduction

The population demographics of the United States are changing. The number of people with limited English proficiency, defined as having English as a second language and possessing limited ability to read, write, speak, and understand the English language, is increasing. Based on the U.S. Census Bureau 2018 data, ∼64 million Americans speak a language other than English at home, an increase of 1.5 million from 2015.^1^ This trend is projected to continue.^2^

In the United States, people with limited English proficiency are at risk for experiencing health care disparities in accessing health care and screenings.^3^ Persons with limited English proficiency perceive poorer patient–physician interaction relative to people who primarily speak English.^4^ Compared to those who only speak English, people with limited English proficiency are less likely to have a regular health care provider, have fewer physician visits, and lower rates of screening (e.g., blood pressure, cancer).^3,5–9^ Persons with limited English proficiency may also have other characteristics that affect their ability to access health services, including older age,^10^ low health literacy,^11^ cultural practices that limit questioning health care providers,^12^ and fewer community-support services.^13^

The Affordable Care Act (ACA) passed in 2010 created pathways for insurance that were to be mediated by support services, particularly for persons with limited English proficiency and those with low-health literacy.^14^ The proportion of people uninsured dropped to 8.6% of the overall population, with 21.3 million more people insured in 2015 than in 2010.^15,16^ While insurance coverage has increased nationally, research about the extent to which persons with limited English proficiency attained insurance after the implementation of the ACA is scant. One recent study showed that significant gains in insurance covered occurred after ACA for those with high and limited English proficiency, narrowing the health disparities gap.^17^ However, the role of primary language spoken for those with limited English proficiency was not explored. Using a large, nationally representative sample, our study sought to fill this research gap by evaluating the extent to which insurance attainment and usual source of health care varied across groups defined by limited English proficiency and language spoken at home. Given the rapidly changing demographics of the population of the United States, understanding the extent to which adults with limited English proficiency have insurance and access to care in the post-ACA era is imperative.

## Methods

The data were collected through a national survey which was approved by Westat Institutional Review Board by the Office for Protection from Research Risk. The data were anonymized and deidentified. Data were then compiled and released as open-source data and are available to the public. This secondary analysis of publicly available data did not require institutional board review at the University of Massachusetts Medical School.

### Study design and data source

We conducted a cross-sectional study. We used data from the 2016 Medical Expenditure Panel Survey (MEPS), a nationally representative sample of the civilian noninstitutionalized population of the United States.^18^ The Agency for Healthcare Research and Quality and the Center for Disease Control sponsored data collection for MEPS. Annual phone interviews were conducted with randomly selected persons for household reports. Medical providers, pharmacies, and hospitals were contacted based on information provided by household participants.^19^

#### Sample

MEPS 2016 included 34,655 participants. We excluded 1721 participants whose responses were coded as “refused,” “don't know,” “not ascertained,” and “Inapplicable” on key variables, including English language proficiency (*n*=44), education (*n*=313), usual care provider (*n*=1350), and citizenship (*n*=14). From the 32,934 participants with valid responses to key variables, we included 24,099 respondents ≥18 years of age (weighted to represent 240,035,048 adults).^20^

#### Operational definition of limited English proficiency

We used questions in the MEPS Household Component survey to operationally define limited English proficiency/language categories. We first classified participants according to responses to the following questions: “How well do you speak English? Would you say… Very well; well; Not well; Not at all?” We categorized participants as: (1) having limited English proficiency (not well; not at all); English proficient (well; very well); and English only (not applicable).^5^ Next, we classified people based on their response to: “What language do you speak at home? Would you say… English, Spanish, Other.” Using these two recoded variables, we created a variable with five categories: (1) Spanish speaking, with limited English proficiency, (2) other language speaking, with limited English proficiency, (3) Spanish speaking, English proficient, (4) other language speaking, English proficient, and (5) English only.

#### Operational definition of insurance and access

Health insurance coverage and having a usual care provider were based on self-report. MEPS included the following question: Do you have health insurance coverage? If yes, respondents indicated whether or not the insurance was public (e.g., Medicaid, Medicare) or private. We classified people as having private health insurance, public health insurance, or no health insurance. The MEPS questionnaire included the following questions: Do you have a usual source of care? (yes/no). MEPS defines the usual source of care as “the particular medical professional, doctor's office, clinic, health center, or other place where a person would usually go if sick or in need of advice about his or her health.”^18^ Conceptually, we were interested in lack of usual care provider. Consistent with this conceptual definition, we created a binary variable “lack of usual care” which was equal to one if they answered “no” to the usual care question and equal to zero if they answered “yes.” Questions regarding health insurance and a usual source of health care have demonstrated reliability in assessing access to care in adult populations.^21,22^

#### Covariates

Personal characteristics included gender, age, race/ethnicity (Latino/Hispanic, non-Hispanic Black, non-Hispanic Asian, non-Hispanic White), education status (no degree, high school diploma (or equivalency), some college, or beyond), born in the United States (yes, no), born in the United States (yes, no) marital status (married, single, never married), and family income as a percentage of the poverty line (poor, near poor, low income, middle income, high income). Education status was consolidated into three categories: no degree, high school diploma (or equivalency), and some college or greater. Racial categorical data included persons that identified as mixed race within the dominant racial category (e.g., Asian, Black, White). Ethnicity data coded as mixed race were included in the ethnic category of Hispanic if they were both mixed race and Hispanic (e.g., Asian-Hispanic, Black-Hispanic, White-Hispanic).

#### Data analysis

We applied the statistical techniques specified by MEPS to generate population-based estimates.^23^ We used descriptive statistics to characterize the population according to English language proficiency, including mean and standard deviation for continuous variables and percentages for categorical variables. The first outcome of interest had three categories. As such, we used multinomial logistic modeling to evaluate the relationship between limited English proficiency (primary determinant) and insurance coverage (outcome variable). We considered private insurance as the referent group for the outcome variable. We considered English only as the referent group for the primary determinant. We created a logistic model to evaluate the association between limited English proficiency and having a usual care provider. We used the same modeling approach for the logistic and the multinomial models. We first adjusted the model for age, sex, and marital status. We then included terms for income level, education, and born in the United States as a proxy for citizenship. From the models, we derived odds ratios (ORs) and 95% confidence intervals (CIs). The analyses were then stratified by age group (<65 and ≥65 years) because people ≥65 years are eligible for Medicare and the patterns of health care access and use may be substantially different. In the older age group, we were unable to estimate uninsured ORs because there were fewer than five people in some cells. We used STATA version 16.0 (College Station, TX, USA) for all analyses.

## Results

### Population characteristics

Table 1 shows that 4.2% of adults in the United States were Spanish speaking, with limited English proficiency, and 1.1% spoke another language, with limited English proficiency. Persons who primary speak a language other than English at home, but who are proficient in English represent 17.1% of the overall population (Spanish, English proficient: 9.6%; other language, English proficient: 7.5%). People with limited English proficiency were the most likely to have no degree (Spanish, limited English proficiency: 65.5%, other language, limited English proficiency: 40.0%, English only: 9.7%). The distribution of income varied by limited English proficiency, with those with limited English proficiency the most likely to be in the Poor income group (Spanish, limited English proficiency: 24.8%, other language, limited English proficiency: 27.2%, English only: 10.0%).

**Table 1.** Characteristics of Adults in the United States, by Language Proficiency (Medical Expenditure Survey Panel 2016)

	Limited English proficiency		English proficient		
	Spanish speaking	Other languages	Spanish	Other languages	English Only
*n*	2,436	393	3,863	2,026	15,381
Weighted, *n*	10,057,469	2,688,393	22,947,351	18,098,643	186,243,194
Age (years), mean	48.6	60.6	39.4	42.6	48.7
	Percentages^a^				
Women	54.5	63.4	49.3	49.0	52.0
Race/ethnicity					
Hispanic	98.6	2.6	85.9	3.2	4.2
Non-Hispanic White	1.2	1.5	11.0	32.5	77.4
Non-Hispanic Black	0.2	8.7	1.8	11.1	13.5
Non-Hispanic Asian	0.0	72.6	0.4	49.3	1.5
Non-Hispanic multirace or other	0.0	0.5	0.9	3.9	3.3
Marital status					
Married	58.2	74.9	46.6	61.9	52.1
Divorced, widowed, separated	17.9	17.2	15.2	9.1	20.6
Never married	23.9	7.8	38.2	29.0	27.3
Education					
No degree	65.5	40.0	20.4	8.5	9.7
High school diploma	26.0	32.6	50.1	32.5	49.5
Some college or beyond	8.5	27.4	29.5	59.0	40.7
Income					
Poor	24.8	27.2	12.6	8.5	10.0
Near poor	9.4	6.8	5.2	2.4	3.5
Low income	23.7	15.3	15.6	8.4	11.4
Middle income	33.5	27.6	35.4	27.4	28.0
High income	8.5	23.1	31.2	53.3	47.0
Born in the United States	50.4	20.3	58.3	29.1	96.6

^a^ Percentages may not total to 100% due to rounding.

### Association between limited English proficiency and health insurance

**FIG. 1.**
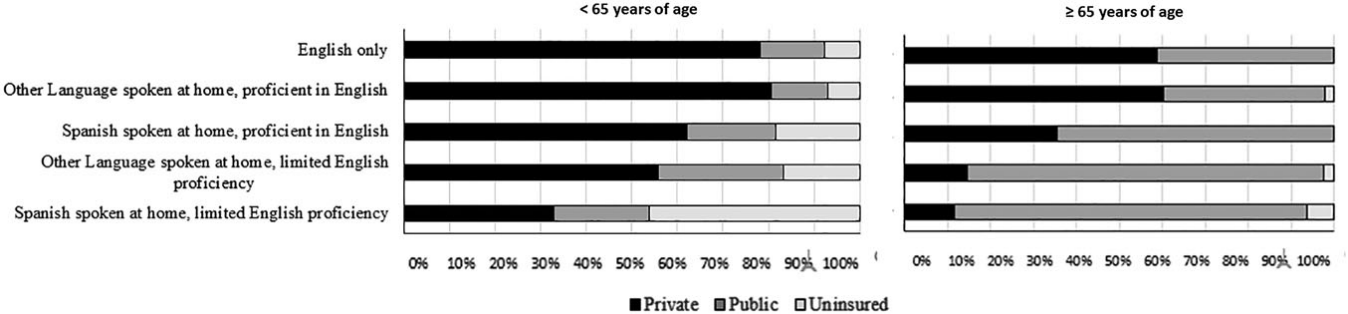
Percent of health insurance type by language proficiency group for adults <65 years of age and ≥65 years of age in the United States, 2016.

The distribution of health insurance by limited English proficiency is shown stratified by age group (Fig. 1). Table 2 shows that relative to adults who speak English only, adults with limited English proficiency in the United States had increased odds of receiving public insurance (fully adjusted OR [Spanish speaking]=1.6, 95% CI: 1.3–2.1; fully adjusted OR [other language]=2.9, 95% CI: 1.8–4.5) or being uninsured (fully adjusted OR [Spanish speaking]=4.7, 95% CI: 3.6–6.0; fully adjusted OR [other language]=1.8, 95% CI: 1.0–3.1). Adults who spoke Spanish or another language and were proficient in English had increased odds of receiving public insurance relative to adults who only spoke English (fully adjusted OR [Spanish]=1.6, 95% CI: 1.4–1.9; fully adjusted OR [other language]=1.3, 95% CI: 1.0–1.6). While Spanish speaking adults proficient in English had increased odds of being uninsured (fully adjusted OR=1.9, 95% CI: 1.5–2.3), adults who spoke another language and were proficient in English had decreased odds of being uninsured (fully adjusted OR=0.7, 95% CI: 0.5–1.0). The patterns in the overall analysis were similar to the patterns in people <65 years of age. For those ≥65 years, those with limited English proficiency had higher odds of being covered only by public insurance (vs. any private insurance) than those who only spoke English (Spanish: aOR: 3.6; 95% CI: 2.2–6.1; Other languages: aOR: 4.1; 95% CI: 1.9–8.9). The full model results are available in Appendix Table A1.

**Table 2.** Association Between Language Proficiency and Public Only or Lack of Insurance Coverage (2016)

	Adjusted for age, sex, and marital status		Adjusted for age, sex, marital status, family income, citizenship status, and education	
	OR	95% CI	OR	95% CI
Overall				
Public only versus any private insurance				
Spanish, limited English	4.57	3.79–5.51	1.60	1.23–2.07
Other language, limited English	5.46	3.85–7.73	2.86	1.83–4.46
Spanish, English proficient	1.89	1.63–2.20	1.61	1.37–1.88
Other language, English proficient	1.02	0.84–1.25	1.27	1.00–1.60
English only	1.0	—	1.0	—
Uninsured versus any private insurance				
Spanish, limited English	20.53	14.50–21.76	4.67	3.64–5.99
Other language, limited English	5.69	2.82–7.55	1.81	1.05–3.13
Spanish, English proficient	2.97	2.43–3.54	1.87	1.54–2.28
Other language, English proficient	0.97	0.68–1.32	0.71	0.49–1.02
English only	1.0	—	1.0	—
<65 years of age				
Public only versus any private insurance				
Spanish, limited English	4.52	3.59–5.70	1.41	1.00–1.98
Other language, limited English	4.24	2.82–6.38	2.02	1.18–3.43
Spanish, English proficient	1.76	1.49–2.08	1.55	1.29–1.88
Other language, English proficient	1.0	0.78–1.28	1.49	1.11–2.01
English only	1.0	—	1.0	—
Uninsured versus any private insurance				
Spanish, limited English	17.76	14.50–21.76	4.01	3.06–5.24
Other language, limited English	4.61	2.82–7.55	1.44	0.78–2.67
Spanish, English proficient	2.93	2.43–3.54	1.91	1.56–2.35
Other language, English proficient	0.95	0.68–1.32	0.77	0.53–1.12
English only	1.0	—	1.0	—
≥65 Years of age				
Public only versus any private insurance				
Spanish, limited English	10.10	6.48–15.75	3.64	2.17–6.11
Other language, limited English	8.62	4.47–16.62	4.14	1.91–8.94
Spanish, English proficient	2.66	1.88–3.74	1.87	1.30–2.69
Other language, English proficient	0.98	0.69–1.38	0.78	0.52–1.16
English only	1.0	—	1.0	—

Uninsured versus private insurance (not estimable owing to cells with fewer than five observations).

### Association between limited English proficiency and lack of usual health care provider

Table 3 shows the association between limited English proficiency and lack of usual health care provider. Relative to adults who speak English only, adults with limited English proficiency in the United States had increased odds of lacking a usual health care provider (age, sex, marital status adjusted ORs [Spanish speaking]: 2.9, 95% CI: 2.4–3.3; [Other language]: 2.2, 95% CI: 1.5–3.3). Further adjustment for proxies of socioeconomic positioning, born in the United States, health insurance, and education diminished the ORs (aOR [Spanish speaking]: 1.7; 95% CI: 1.4–2.1; aOR [Other language]: 1.8, 95% CI: 1.2–2.7). Adults who were Spanish speaking, but English proficient had increased odds of lacking a usual health care provider relative to English only adults (aOR: 1.2, 95% CI: 1.0–1.4). The patterns in the overall analysis were like patterns in people <65 years of age. For those ≥65 years, those with limited English proficiency had higher odds of being covered only by public insurance (vs. any private insurance) than those who only spoke English (Spanish: aOR: 2.3; 95% CI: 1.1–4.7). The full model results are available in Appendix Table A2.

**Table 3.** Association Between Language Proficiency and Lack of Usual Care Provider (2016)

	Adjusted for age, sex, and marital status		Adjusted for age, sex, marital status, family income, citizenship status, insurance, and education	
	OR	95% CI	OR	95% CI
Overall (reference group English only)				
Spanish, limited English proficiency	2.83	2.41–3.32	1.70	1.37–2.11
Other language, limited English proficiency	2.20	1.47–3.26	1.76	1.15–2.67
Spanish, English proficient	1.43	1.23–1.65	1.19	1.02–1.39
Other language, English proficient	1.16	0.97–1.40	1.02	0.83–1.26
<65 Years of age (reference group English only)				
Spanish, limited English proficiency	2.79	2.35–3.31	1.64	1.31–2.05
Other language, limited English proficiency	2.37	1.51–3.72	1.84	1.16–2.93
Spanish, English proficient	1.42	1.22–1.65	1.18	1.01–1.38
Other language, English proficient	1.11	0.92–1.34	0.97	0.79–1.18
≥65 Years of age (reference group English only)				
Spanish, limited English proficiency	2.58	1.64–4.08	2.26	1.10–4.65
Other language, limited English proficiency	2.13	0.87–5.18	2.11	0.74–6.01
Spanish, English proficient	1.64	0.91–2.93	1.65	0.89–3.06
Other language, English proficient	2.38	1.29–4.40	2.50	1.15–5.43

## Discussion

The objective of this cross-sectional study was to provide a description of access to health insurance and lack of usual source of health care for people with limited English proficiency after the implementation of the ACA. Our data show that the percentage of people with public health insurance was higher among those with limited English proficiency relative to adults who speak only English. In the post-ACA era, relative to those who spoke English only, the percent uninsured among Spanish speakers was higher regardless of English language proficiency, while the percent uninsured among those with limited English proficiency who spoke another language was lower for adults <65 years of age. Fewer persons with limited English proficiency and Spanish speaking adults had a usual care provider relative to people who only spoke English. Spanish speaking older adults with limited English proficiency remained at risk for lacking a usual health care provider.

We found that an estimated 40% of those uninsured were Spanish speakers; yet, Spanish speakers comprise only ∼14% of the population. Being uninsured has been associated with negative health outcomes and premature death.^24^ Concerns were raised about the availability to support people with limited English proficiency attempting to obtain insurance through the ACA,^14^ particularly language accessible materials and linguistically diverse support staff. The type of insurance someone possesses significantly affects the type of health care one receives,^21^ and people with limited English proficiency experience disparate care due to language accessibility when seeking services.^5,6^ Furthermore, regardless of language (Spanish or other), fewer adults with limited English proficiency reported having a usual care provider after adjusting for insurance coverage. There may be barriers to insurance access and having a usual care provider in the era after the ACA implementation that adversely impacts Spanish speaking adults and those with limited English proficiency. The MEPS data lacked specific information regarding potential barriers. Further research must explore what these barriers are and identify solutions to address them.

### Health equity implications

We found that Spanish speakers, regardless of English proficiency, and those who spoke another language with limited English proficiency were more likely to be uninsured and more likely to lack a usual health care provider than those who spoke English only. Having a higher level of education is associated with increased likelihood of insurance coverage.^16^ Differences remained after adjustment for education and other socioeconomic variables. Language accessibility is a potential contributing factor to having a usual care provider. Pathways for increased access to usual care providers are needed. For example, some insurance companies are publishing the languages spoken by health care providers.^25^ While there remains a lack of health care providers for persons with limited English proficiency,^26^ telemedicine allows for interpreter services to be available in almost any location in the United States, provided that the location has stable internet connection or telephone service.^27,28^

### Study strengths and limitations

This study provides an important overview of access to insurance and lack of usual health care providers among adults with varying degrees of English language proficiency in the post ACA era. Using MEPS, we were able to provide national estimates of vulnerable populations in need of additional support. Despite the unique nationally representative dataset provided by MEPS, there are limitations to consider. First, the MEPS interviews are conducted over the phone which may raise concerns about translation errors when communicating with persons who are not native English speakers. Fortunately, MEPS has a team of interviewers able to conduct interviews in multiple languages.^19^ MEPS also presents interviewers with questions that can be read in multiple languages so that interviewers do not have to translate the questions and, thus, improve the accuracy of interviews not conducted in English.^29^ Second, MEPS only determined whether English or Spanish was spoken at home.^30^ Our analysis used data from 2016. We selected 2016 because of uncertainty about ACA continuity after the 2016 presidential election in the United States. We feared that ACA uncertainty could have impacted insurance utilization. Despite these limitations, our study contributes timely, much needed data about language accessibility, access to insurance coverage, and source of usual health care.

## Conclusions

Our findings show that a greater percent of adults lacking English language proficiency were uninsured and lacked a usual care provider. Spanish-speaking persons appeared to be more vulnerable to being uninsured despite increased access to insurance provided by the ACA. Persons with the lowest level of English proficiency were the most likely to lack a usual care provider even when adjusting for insurance coverage. More research is needed to investigate the barriers and facilitators of insurance coverage in the era post ACA for limited English proficient populations and those who speak Spanish.

## Abbreviations Used

ACA: Affordable Care Act
CI: confidence interval
MEPS: Medical Expenditure Panel Survey
OR: odds ratio

## Appendix

**Appendix Table A1.** Other Variables in the Full Models Shown in Table 2

	Versus any private insurance
Overall	Public only: OR (95% CIs)
Age (years)	1.04 (1.04–1.05)
Female	1.16 (1.07–1.26)
Marital status (married is the reference)	
Widowed/divorced/separated	1.56 (1.35–1.81)
Never	2.60 (2.29–2.93)
Born in the United States	1.04 (0.88–1.23)
Income level (poor is reference)	
Near poor	0.60 (0.45–0.79)
Low income	0.37 (0.31–0.44)
Middle income	0.12 (0.10–0.14)
High income	0.06 (0.05–0.07)
Education (<high school is reference)	
High school	0.50 (0.44–0.56)
≥High school	0.30 (0.25–0.39)
<65 Years	
Age (years)	1.01 (1.00–1.02)
Female	1.34 (1.20–1.48)
Marital status (married is the reference)	
Widowed/divorced/separated	2.19 (1.81–2.65)
Never	2.36 (1.98–2.81)
Born in the United States	1.14 (0.90–1.44)
Income level (poor is reference)	
Near poor	0.63 (0.47–0.83)
Low income	0.35 (0.29–0.42)
Middle income	0.08 (0.07–0.10)
High income	0.02 (0.02–0.03)
Education (<high school is reference)	
High school	0.49 (0.43–0.57)
≥High school	0.23 (0.19–0.27)
≥65 Years	
Age (years)	1.01 (1.00–1.02)
Female	0.98 (0.85–1.12)
Marital status (married is the reference)	
Widowed/divorced/separated	1.30 (1.06–1.61)
Never	1.58 (1.04–2.38)
Born in the United States	0.69 (0.51–0.92)
Income level (poor is reference)	
Near poor	0.82 (0.53–1.26)
Low income	0.84 (0.61–1.15)
Middle income	0.48 (0.36–0.64)
High income	0.34 (0.25–0.46)
Education (<high school is reference)	
High school	0.53 (0.41–0.69)
≥High school	0.43 (0.32–0.58)

**Appendix Table A2.** Other Variables in the Full Models Shown in Table 3

	Adjusted for age, sex, marital status, family income, citizenship status, insurance, and education	
Overall	OR	95% CI
Age (years)	0.97	0.96–0.97
Female	0.60	0.56–0.64
Marital status (married is the reference)		
Widowed/divorced/separated	1.35	1.19–1.55
Never	1.24	1.10–1.40
Born in the United States	0.80	0.70–0.92
Income level (poor is reference)		
Near poor	0.83	0.68–1.01
Low income	0.93	0.80–1.07
Middle income	0.75	0.65–0.87
High income	0.66	0.57–0.76
Insurance (any private is reference)		
Public only	0.78	0.69–0.89
Uninsured	3.13	2.72–3.59
Education (<high school is reference)		
High school	1.44	1.26–1.64
≥High school	1.46	1.25–1.69
<65 years		
Age (years)	0.97	0.97–0.98
Female	0.59	0.54–0.63
Marital status (married is the reference)		
Widowed/divorced/separated	1.28	1.11–1.49
Never	1.30	1.15–1.47
Born in the United States	0.80	0.69–0.92
Income level (poor is reference)		
Near poor	0.80	0.65–0.98
Low income	0.91	0.79–1.06
Middle income	0.73	0.63–0.85
High income	0.63	0.54–0.74
Insurance (any private is reference)		
Public only	0.76	0.69–0.92
Uninsured	3.01	2.62–3.47
Education (<high school is reference)		
High school	1.47	1.29–1.69
≥High school	1.52	1.30–1.78
≥65 Years		
Age (years)	0.97	0.95–0.99
Female	0.75	0.58–0.97
Marital status (married is the reference)		
Widowed/divorced/separated	1.66	1.19–2.31
Never	0.94	0.50–1.75
Born in the United States	1.11	0.64–1.91
Income level (poor is reference)		
Near poor	1.17	0.66–2.07
Low income	1.11	0.65–1.88
Middle income	0.99	0.62–1.59
High income	0.98	0.59–1.61
Insurance (any private is reference)		
Public only	1.05	0.77–1.43
Uninsured	5.99	2.26–15.88
Education (<high school is reference)		
High school	1.05	0.71–1.55
≥High school	0.89	0.58–1.38

CI, confidence interval; OR, odds ratio.
